# Supplementary material for: A high-quality genome assembly highlights the evolutionary history of the great bustard (Otis tarda, Otidiformes)
Source: Commun Biol. 2023 Jul 18;6:746. doi: 10.1038/s42003-023-05137-x (PMC10354230; doi:10.1038/s42003-023-05137-x)
Supplement: Supplementary file 2 — Supplementary Information [file 42003_2023_5137_MOESM2_ESM.pdf]

Supplementary Information

Supplementary for: A high-quality genome assembly highlights the evolutionary history of the great bustard (*Otis tarda*, Otidiformes)

Haoran Luo[1,2, #], Xinrui Jiang[1, #], Boping Li[3, #], Jiahong Wu[1], Jiexin Shen[1], Zaoxu Xu[3], Xiaoping Zhou[2], Minghao Hou[3], Zhen Huang[4, 5,*], Xiaobin Ou[3, *], Luohao Xu[1, *]

Correspondence: Z.H. zhuang@fjnu.edu.cn, X.O. xbou@zju.edu.cn, L.X. luohaox@swu.edu.cn

Supplementary Table 1-11

Supplementary Figure 1-9

Supplementary tables

Supplementary table 1. Sequencing data summary

Sequencing item	Total number of sequenced bases (Gb)	Coverage (X)
ONT ultra-long	134.14	112.72x
Short reads	73.34	61x
Hi-C	58.62	49.2x

Supplementary table 2. Genome assembly statistics

Statistic term	Result
Contig Total length (bp)	1,198,250,872
No. of contigs	129
Longest contig (bp)	131,880,296
Contig N50 (bp)	40,962,436
Contig L50	8
Contig N90 (bp)	8,651,169
Contig L90	31
GC content	42.94 %
No. of Scaffold	117
Scaffold Total length	1,198,259,872
Longest Scaffold	216,409,348
Scaffold N50	82,831,862
Anchored length	1,171,228,342
Anchored chromosome	39+Z

Supplementary table 3. Telomeric repeats distribution

Chromosome	Start	End	Chromosome length	Telomere length
Chr1	298	3638	216409348	3341
Chr4a	23222495	23228192	23239934	5698
Chr6	39674897	39675196	39679499	300
Chr7	42611259	42611619	42621419	361
Chr8	34274625	34274867	34277949	243
Chr10	23456930	23461302	23461302	4373
Chr11	22596384	22597987	22597989	1604
Chr12	23973482	23978844	23978844	5363
Chr13	20915227	20916404	20916404	1178
Chr14	9	6164	18728488	6156
Chr17	12545486	12545559	12545563	74
Chr18	13507851	13509446	13511118	1596
Chr20	340793	340990	17061717	198
Chr20	17056270	17057464	17061717	1195
Chr21	2262	4093	9945557	1832
Chr23	8641334	8641884	8651169	551
Chr24	7233430	7233783	7241344	354
Chr26	5284	6116	7317679	833
Chr27	8000857	8009591	8009591	8735
Chr28	3188	7000	6272520	3813
Chr36	1560405	1560578	1624084	174

Supplementary table 4. Repeat elements annotation

Type	Length (bp)	Percentage of genome
Type I: Retroelements	81,307,069	6.79 %
SINE	1,141,104	0.1%
LINE	59,715,870	4.98%
LTR	20,450,095	1.71%
Type II: DNA transposons	27,092,422	2.26%
Type III: Tandem repeat	32,472,544	2.71%
Satellites	18,475,858	1.54 %
Simple repeats	11,205,782	0.94%
Low complexity	2,790,904	0.23 %
Type IV: Small RNA	73,926	0.01%

Unclassified: 3.29%

Supplementary table 5. GC content and Methylation levels

Chr	Chr length (bp)	GC%	Methylation Level	Methylated CpG site (%)	Methylated Density (50kb-window)
1	216409348	40.77	0.5937	0.877396109	399.171
2	164758815	40.44	0.5959	0.879942686	393.738
3	126661284	41.01	0.6007	0.87612231	418.381
4	82831862	40.56	0.6026	0.879262973	423.176
4a	23239934	44.14	0.6083	0.857710821	595.941
5	71358559	41.89	0.5924	0.861680502	456.72
6	39679499	41.93	0.5992	0.867906082	484.443
7	42621419	41.84	0.6123	0.873357327	494.472
8	34277949	42.52	0.6050	0.864929136	535.328
9	28240577	43.30	0.6063	0.862677839	555.417
10	23461302	43.82	0.5949	0.85064629	574.29
11	22597989	43.14	0.6146	0.8612378	571.802
12	23978844	44.07	0.6087	0.862232672	602.013
13	20916404	45.25	0.5667	0.833927806	624.734
14	18728488	45.69	0.5946	0.836141664	637.313
15	16151846	46.25	0.5780	0.827455574	655.347
16	1101586	61.01	0.5767	0.758301887	962.318
17	12545563	48.91	0.5915	0.827394342	711.48
18	13511118	47.83	0.5796	0.82161188	702.267
19	12724254	47.74	0.6090	0.837419554	763.197
20	17061717	46.66	0.5799	0.831959238	667.551
21	9945557	48.65	0.6067	0.831632589	795.01
22	5822784	50.20	0.5835	0.805588164	811.233
23	8651169	51.89	0.5779	0.801994433	880.503

24	7241344	49.95	0.6030	0.829883425	797.396
25	3644646	59.12	0.5561	0.733301402	945.5
26	7317679	53.09	0.5628	0.789320618	926.137
27	8009591	53.67	0.5496	0.767043367	867.75
28	6272520	54.73	0.5681	0.777525168	951.656
29	795849	66.94	0.4784	0.661041928	872.2
30	2112139	62.74	0.5313	0.701159146	951.452
31	1071183	69.77	0.3706	0.560982133	883.428
32	2012326	61.53	0.5575	0.72419541	968.325
33	2898771	61.31	0.4903	0.68883263	924.228
34	681447	60.14	0.5042	0.71522799	772.077
35	1577892	64.01	0.4717	0.66406883	834.065
36	1624084	62.72	0.5048	0.68658801	836.281
37	1605537	64.85	0.4856	0.656247839	902.375
38	2892653	56.79	0.5325	0.762897633	747.772
Z	84192814	40.85	0.6070	0.880046404	405.993

Supplementary table 6. Comparison of percentage of methylated CpG sites (Tukey HSD)

Target type	Compare Type	Adjusted P-values
Dot	Macro	< 0.0001
	Micro	< 0.0001
Marco	Micro	0.002

Supplementary table 7. Comparison of CpG site density (Tukey HSD)

Target type	Compare Type	Adjusted P-values
Dot	Macro	< 0.0001
	Micro	< 0.0001
Marco	Micro	0.025

Supplementary table 8. Comparison of chromosome-wide methylation levels (Tukey HSD)

Target type	Compare Type	Adjusted P-values
Dot	Macro	2.57E-7
	Micro	3.72E-6
Marco	Micro	0.2194

Supplementary table 9. RNA and RNA sequencing bases quality of 5 tissues

Tissue	A260/A280	A260/230	RIN	*C* (ng/μl)	Q20(B/A)	Q30(B/A)
Leg thigh muscle	1.99	1.27	5.7	451.0	97.62%-97.66%	93.57%-93.63%
Brain	2.03	2.03	8.2	1896.0	97.51%-97.55%	93.40%-93.46%
Heart	1.98	1.65	5.1	670.0	97.46%-97.52%	93.56%-93.65%
Lung	1.95	1.39	8.3	828.0	97.54%-97.58%	93.36%-93.43%
Liver	1.98	1.49	6.5	1905.0	97.31%-97.40%	93.28%-93.43%

C: Concentration. B/A: Bases quality before and after filtered by Fastp program.

Supplementary table 10. Expression levels comparison between PSGs and non-PSGs

Tissue	P-values	Adjusted P-values
Brain	9.551e-018	1.254e-017
Thigh	2.674e-018	4.680e-018
Lung	1.178e-026	6.185e-026
Liver	3.056e-019	8.022e-019
Heart	1.314e-017	1.380e-017

Mann-Whitney test

Supplementary table 11. RT-qPCR primer sequences

Primer	Sequences
OT*Actinβ*-F	5'-TATTGCTGCGCTCGTTGTTG-3'
OT*Actinβ*-R	5'-TGGCCCATACCAACCATCAC-3'
OT*MYF5*-F	5'-AACCAAAGGCTCCCCAAAGT-3'
OT*MYF5*-R	5'-GTCCCGGCAGGTGATAATAGT-3'
OT*MYOD*-F	5'-CTTCTACGATGACCCGTGCT-3'
OT*MYOD*-R	5'-CATGCTCCTCCTCGTGCG-3'

Supplementary Figures

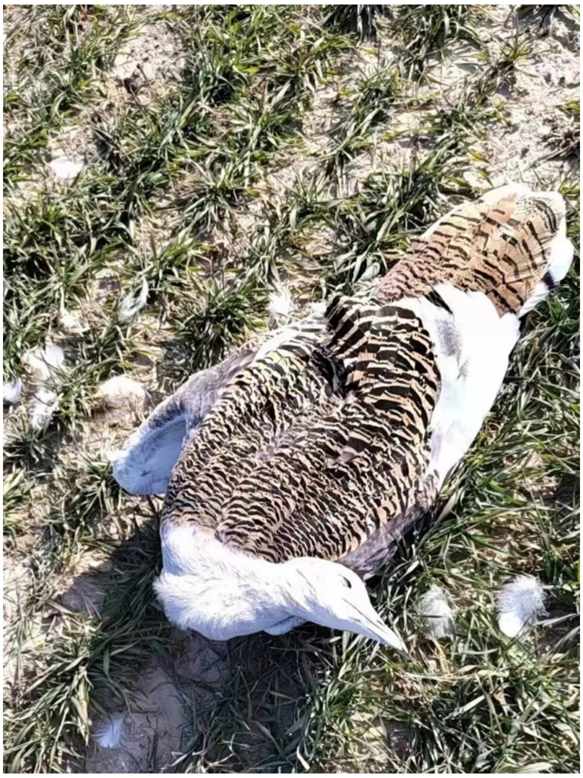

Supplementary Figure 1. A dead male great bustard found at Hesheng Town, Ning County, Qingyang City, Gansu Province, China (35°431255′N, 107°782055′E).

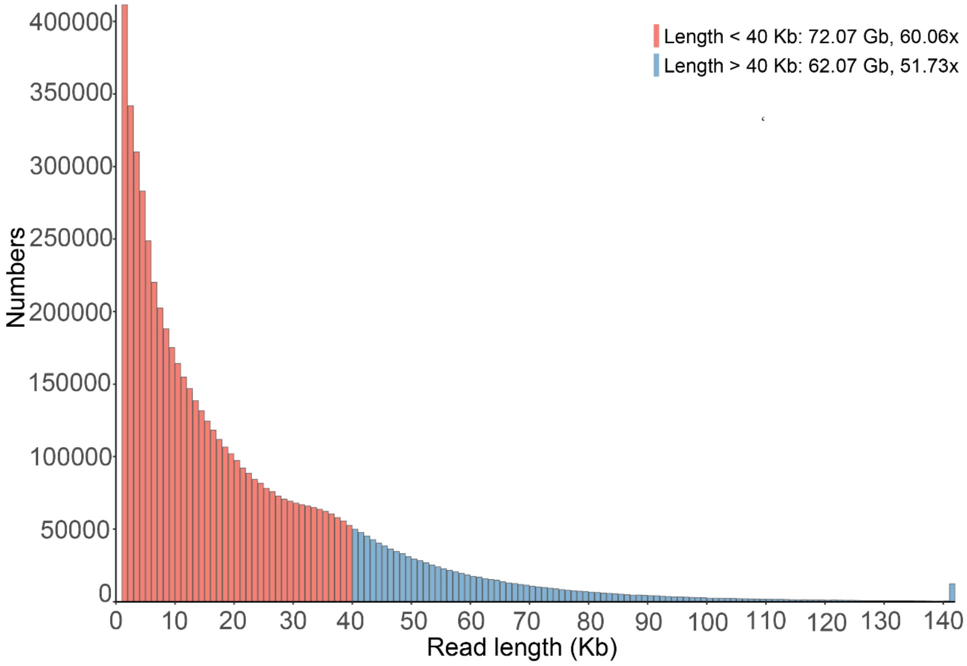

Supplementary Figure 2. Nanopore sequencing read distribution. The N50 of the ultra-long reads reached 37.7 kb. We used read cutoff=40 Kb in the NextDenove. X-axes represent read length and Y-axes represent read count numbers.

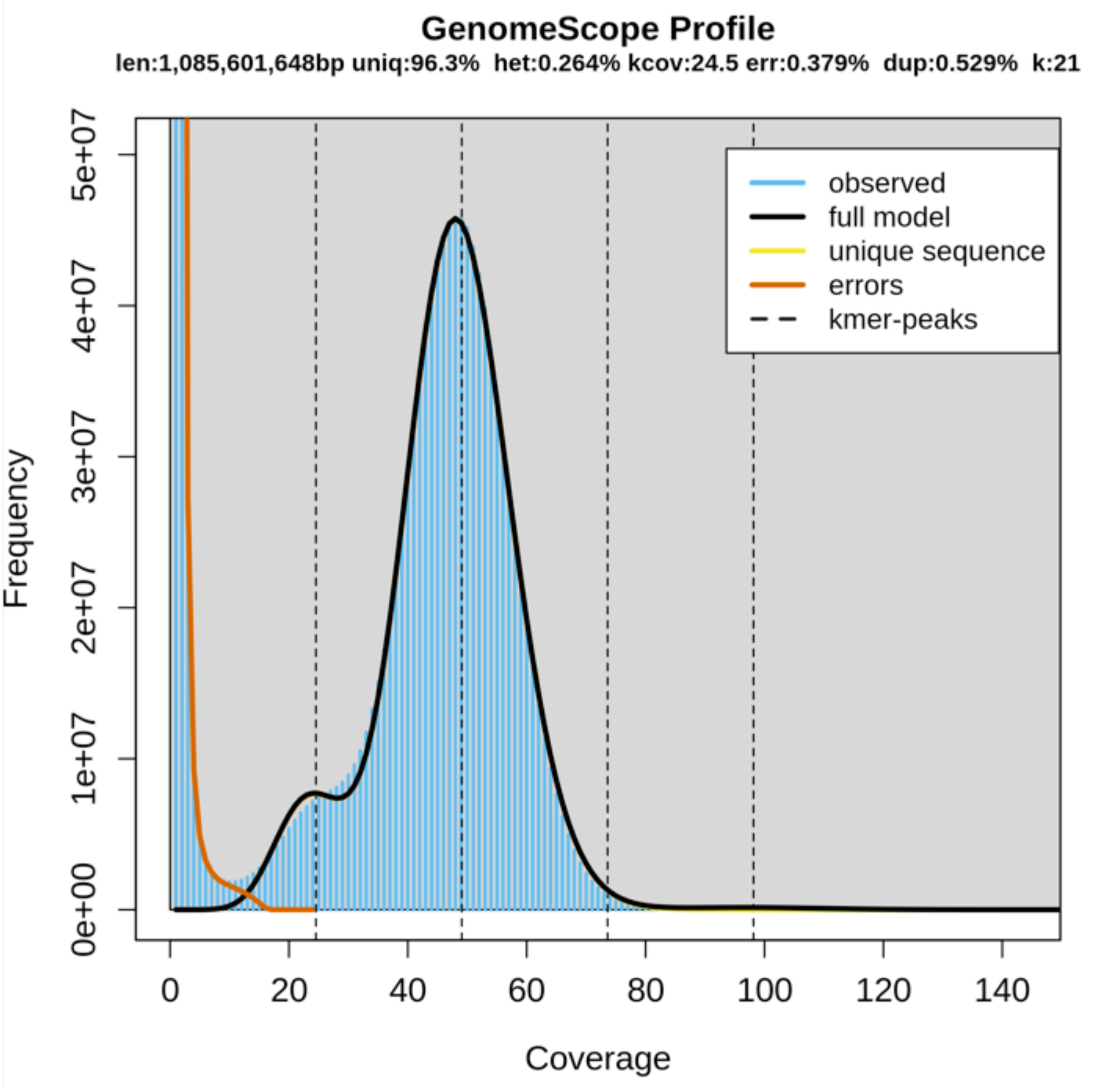

Supplementary Figure 3. Genome survey using short reads. Estimated genome size was evaluated by GenomeScope using 21-Kmer.

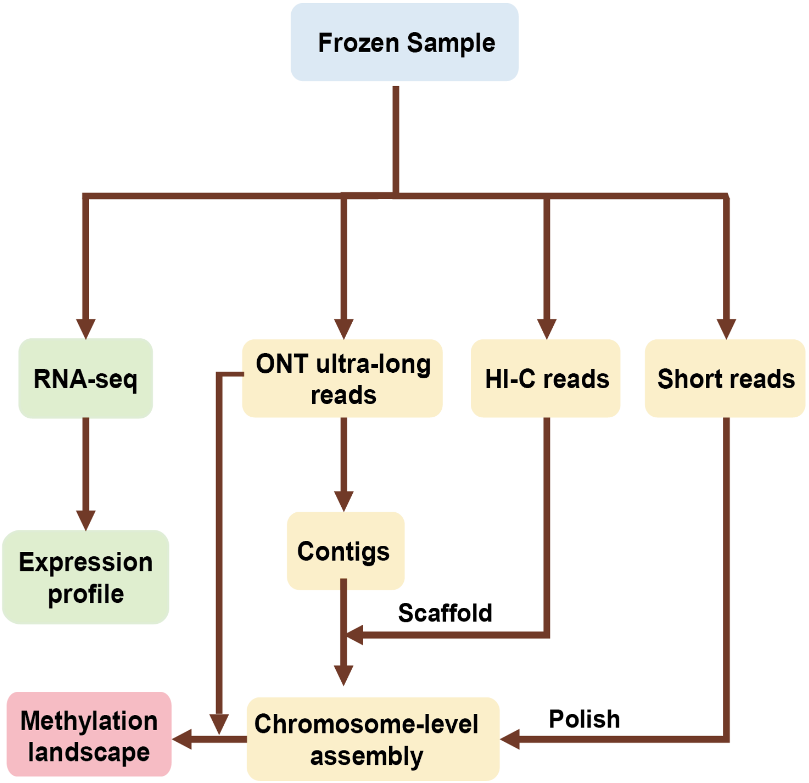

Supplementary Figure 4. Workflow of this study.

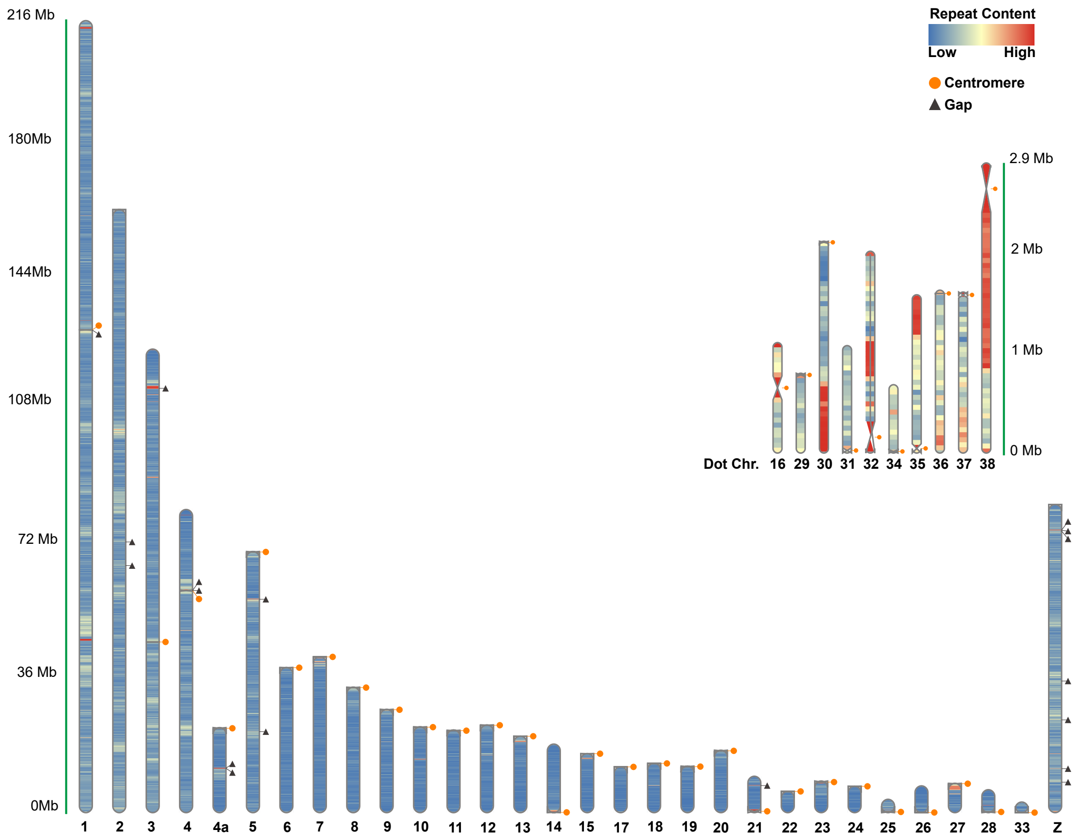

Supplementary Figure 5. Gap and centromere in the assembly. Gap and centromere locations are labeled next to the OTswu assembly chromosomes. Black triangles represent gaps and dot, and orange color represent centromeres.

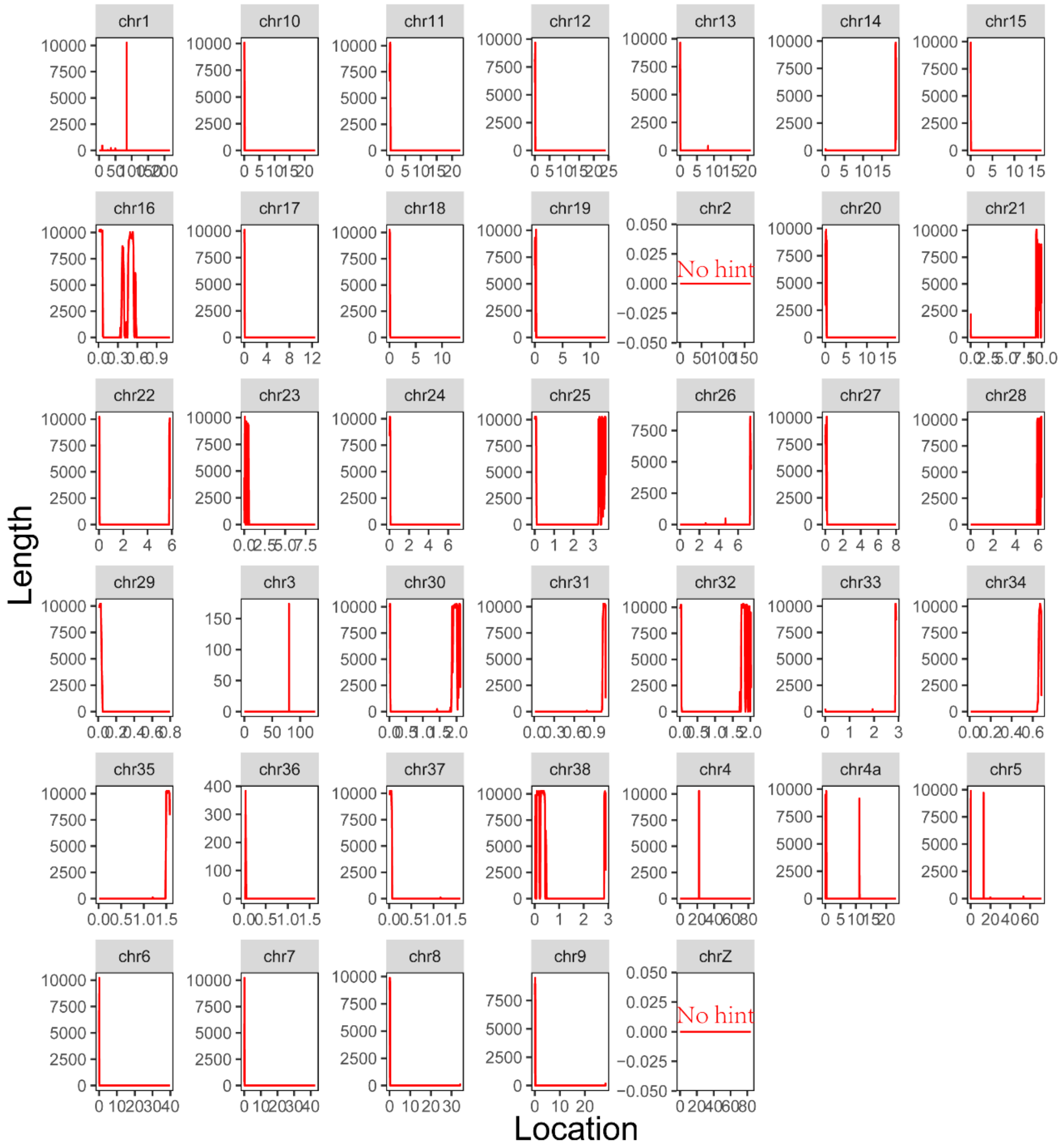

Supplementary Figure 6. The length of putative centromere sequence Cen191 on each chromosome. X-axis representx location in the chromosome (Mb), Y-axis represents the length of Cen191.

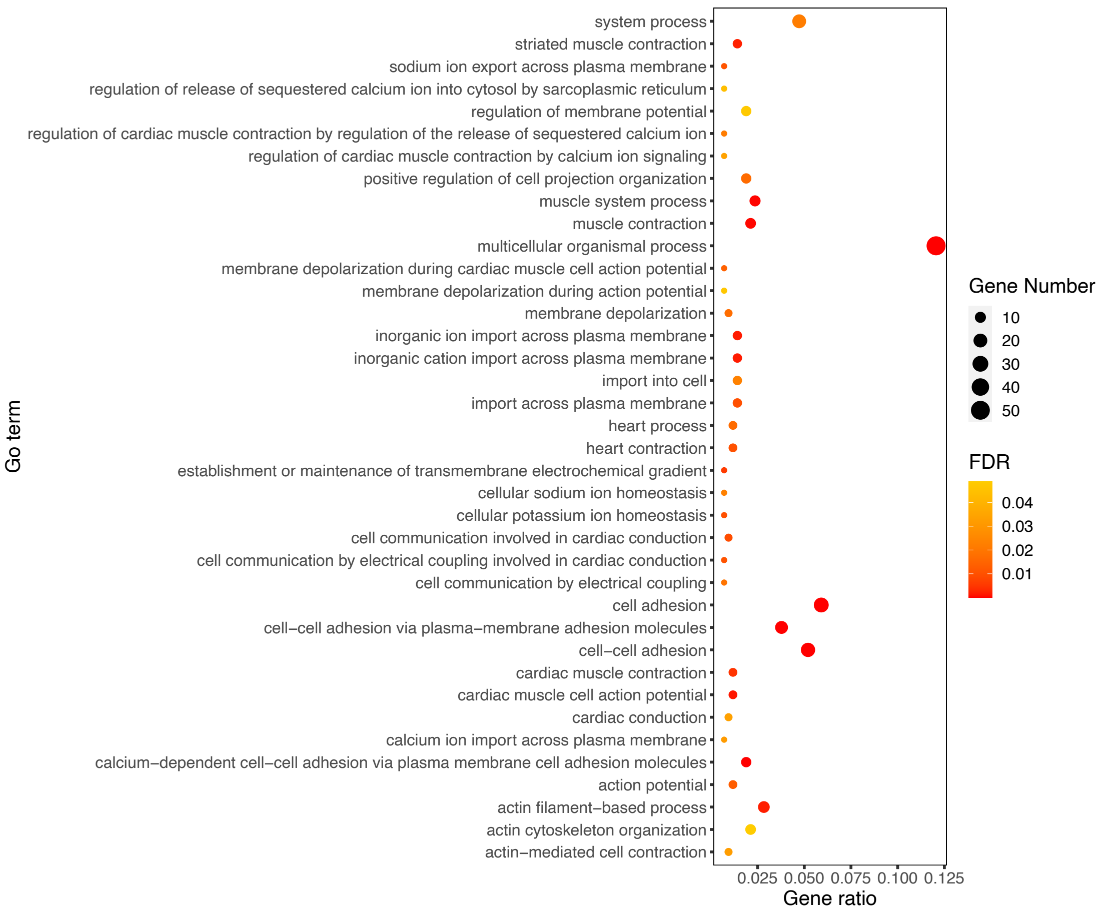

Supplementary Figure 7. GO enrichment of the expanded gene families. Significantly enriched GO terms are mostly related to powered flight.

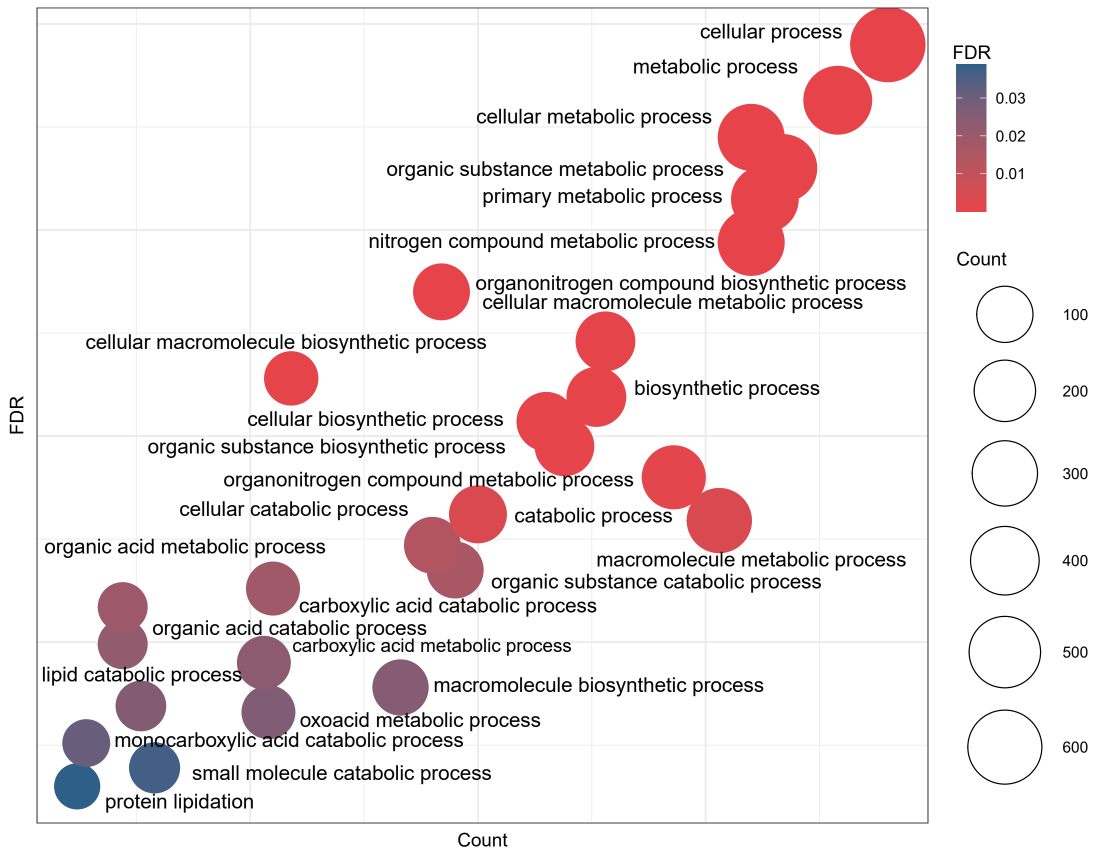

Supplementary Figure 8. Enriched GO terms for PSGs. The sizes of filled circles represent counts of PSGs. The colors of circles indicate GO terms transpose FDR values.

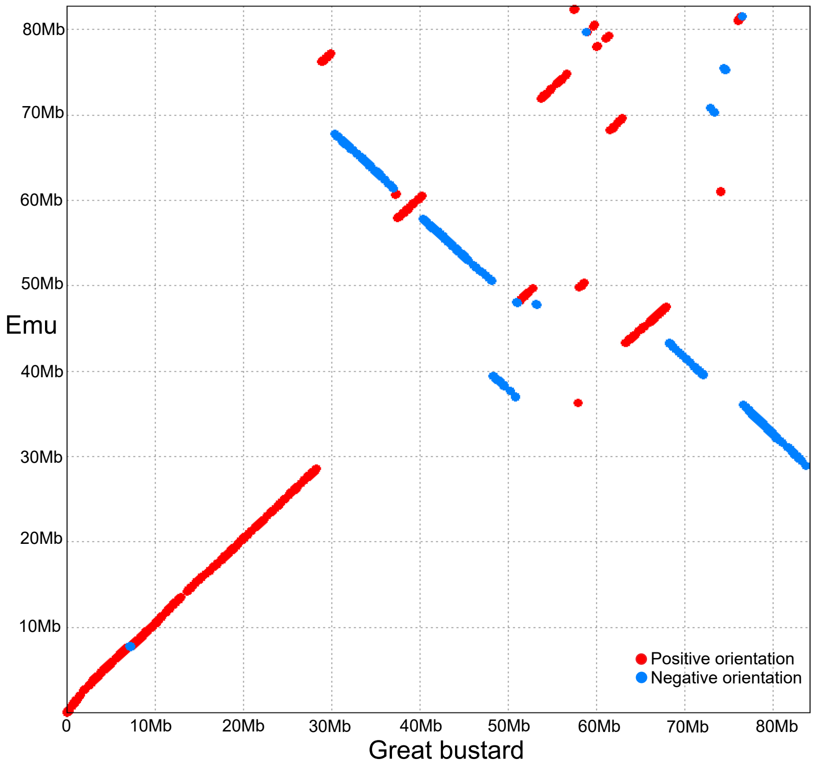

Supplementary Figure 9. Alignment of chromosome Z between emu and great bustard visualized in a dot-plot. Red dots represent alignments with positive orientation; blue dots represent alignments with negative orientation.
