# Supplementary material for: A high-quality genome assembly highlights the evolutionary history of the great bustard (Otis tarda, Otidiformes)
Source: Commun Biol. 2023 Jul 18;6:746. doi: 10.1038/s42003-023-05137-x (PMC10354230; doi:10.1038/s42003-023-05137-x)
Supplement: Supplementary file 3 — Description of Additional Supplementary Files [file 42003_2023_5137_MOESM3_ESM.pdf]

Description of Additional Supplementary Files

File name: Supplementary Data 1

Description: Expanded gene families

File name: Supplementary Data 2

Description: Significantly enriched GO for expanded gene families

File name: Supplementary Data 3

Description: Positively selected genes

File name: Supplementary Data 4

Description: Significantly enriched GO for positively selected genes
