# Supplementary material for: A high-quality genome assembly highlights the evolutionary history of the great bustard (Otis tarda, Otidiformes)
Source: Commun Biol. 2023 Jul 18;6:746. doi: 10.1038/s42003-023-05137-x (PMC10354230; doi:10.1038/s42003-023-05137-x)
Supplement: Supplementary file 8 — Reporting summary [file 42003_2023_5137_MOESM8_ESM.pdf]

nature portfolio

Corresponding author(s): Luohao Xu

Last updated by author(s): 2023-6-29

Reporting Summary

Nature Portfolio wishes to improve the reproducibility of the work that we publish. This form provides structure for consistency and transparency in reporting. For further information on Nature Portfolio policies, see our Editorial Policies and the Editorial Policy Checklist.

Statistics

For all statistical analyses, confirm that the following items are present in the figure legend, table legend, main text, or Methods section.

n/a	Confirmed	
☐	☒	The exact sample size (*n*) for each experimental group/condition, given as a discrete number and unit of measurement
☐	☒	A statement on whether measurements were taken from distinct samples or whether the same sample was measured repeatedly
☐	☒	The statistical test(s) used AND whether they are one- or two-sided<br>*Only common tests should be described solely by name; describe more complex techniques in the Methods section.*
☒	☐	A description of all covariates tested
☐	☒	A description of any assumptions or corrections, such as tests of normality and adjustment for multiple comparisons
☐	☒	A full description of the statistical parameters including central tendency (e.g. means) or other basic estimates (e.g. regression coefficient) AND variation (e.g. standard deviation) or associated estimates of uncertainty (e.g. confidence intervals)
☐	☒	For null hypothesis testing, the test statistic (e.g. *F*, *t*, *r*) with confidence intervals, effect sizes, degrees of freedom and *P* value noted<br>*Give P values as exact values whenever suitable.*
☐	☒	For Bayesian analysis, information on the choice of priors and Markov chain Monte Carlo settings
☒	☐	For hierarchical and complex designs, identification of the appropriate level for tests and full reporting of outcomes
☐	☒	Estimates of effect sizes (e.g. Cohen's *d*, Pearson's *r*), indicating how they were calculated

Our web collection on statistics for biologists contains articles on many of the points above.

Software and code

Policy information about availability of computer code

Data collection	Guppy (v5.0), Nextdenovo (v2.5.0), Nextpolish (v1.4.0), Pilon (v1.24), BUSCO (V5.2.2), Juicer (v1.6)+3D-DNA (v180419), Trimmomatic (v0.39), HISAT2 (v2.1.0), StringTie (v2.0), TransDecoder (v5.5.0), EDTA (v2.0.1), TRF (v4.09), RepeatModeler (v2.0.1), pyTanFinder, CD-hit (v4.8.1), RepeatMasker (v4.1.2-p1), Genome threader (v1.7.1), Exonerate (v2.4.0), Maker3, AUGUSTUS (v3.4.0), GeneID (v1.40), SNAP (v2006728), TRINITY (v2.8.5), EVM (v1.1.1), PASA (v2.5.2), Nanopolish (v1.4.0), OrthoFinder2, IQTREE2, ModelFinder, LAST (v1066), MULTIZ (v11.2), TrimAl (v1.2), PAML (v4.9j), Café (v4.2.1), PANTHER (v17.0), ParaAT 2.0, MAFFT (v7.505), HyPhy, PSMC (v0.6.5), MUMmer (v4.0.0), BWA (v0.7.17), Picard (v2.25.0), GATK (v4.2.0.0), Plink (v1.90b4), Bcftools (v1.9), Samtools (v1.9), Bedtools (v2.29.1)
Data analysis	Data analysis softwares are described and referenced in the method section. The custom scripts and plot source data used in this study have been deposited at Github (https://github.com/hrluo93/great-bustard-genome).

For manuscripts utilizing custom algorithms or software that are central to the research but not yet described in published literature, software must be made available to editors and reviewers. We strongly encourage code deposition in a community repository (e.g. GitHub). See the Nature Portfolio guidelines for submitting code & software for further information.

Data

Policy information about availability of data

All manuscripts must include a data availability statement. This statement should provide the following information, where applicable:

- Accession codes, unique identifiers, or web links for publicly available datasets
- A description of any restrictions on data availability
- For clinical datasets or third party data, please ensure that the statement adheres to our policy

The genome assembly data are available at GenBank under the accession number: JAPMTP000000000. Raw sequence reads are deposited in the BioProject PRJNA903785. Gene models are deposited at Github (https://github.com/hrluo93/great-bustard-genome).

Human research participants

Policy information about studies involving human research participants and Sex and Gender in Research.

Reporting on sex and gender	Not involved
Population characteristics	Not involved
Recruitment	Not involved
Ethics oversight	Not involved

Note that full information on the approval of the study protocol must also be provided in the manuscript.

Field-specific reporting

Please select the one below that is the best fit for your research. If you are not sure, read the appropriate sections before making your selection.

☒ Life sciences ☐ Behavioural & social sciences ☐ Ecological, evolutionary & environmental sciences

For a reference copy of the document with all sections, see nature.com/documents/nr-reporting-summary-flat.pdf

Life sciences study design

All studies must disclose on these points even when the disclosure is negative.

Sample size	One sample for genome assembly and one sample for resequencing and RNA-seq.
Data exclusions	No data were excluded.
Replication	Most results are bioinformatics based. We dit RT-qPCR in three replications for each experiment.
Randomization	No random sampling was used in this study.
Blinding	Not relavant.

Reporting for specific materials, systems and methods

We require information from authors about some types of materials, experimental systems and methods used in many studies. Here, indicate whether each material, system or method listed is relevant to your study. If you are not sure if a list item applies to your research, read the appropriate section before selecting a response.

Materials & experimental systems

n/a	Involved in the study
☒	☐ Antibodies
☒	☐ Eukaryotic cell lines
☒	☐ Palaeontology and archaeology
☐	☒ Animals and other organisms
☒	☐ Clinical data
☒	☐ Dual use research of concern

Methods

n/a	Involved in the study
☒	☐ ChIP-seq
☒	☐ Flow cytometry
☒	☐ MRI-based neuroimaging

Animals and other research organisms

Policy information about studies involving animals; ARRIVE guidelines recommended for reporting animal research, and Sex and Gender in Research

Laboratory animals	Not involved
Wild animals	An adult male great bustard was found dead on the 17th of January, 2022, at Hesheng Town, Ning County, Qingyang City, Gansu Province, China (35°431255′N, 107°782055′E). The local temperature was -7 to 4 °C during the day we collected the animal.
Reporting on sex	A male great bustard samples was used genome assembly. Sex-based Z chromosome analysis used a female sample (Re-sequencing).
Field-collected samples	The frozen individual was immediately relocated to the laboratory stored at -80 °C. Five tissues were collected for sequencing, including leg thigh muscle, brain, heart, lung, and liver. Leg thigh muscle sample performed nanopore sequencing and RNA-seq; brain, heart, lung, and liver samples performed RNA-seq.
Ethics oversight	The Institutional Animal Care and Use Committee (IACUC) of Longdong University has approved the animal ethics.

Note that full information on the approval of the study protocol must also be provided in the manuscript.
